# A comparison of the quality of image acquisition between two different sidestream dark field video-microscopes

**DOI:** 10.1007/s10877-020-00514-x

**Published:** 2020-05-06

**Authors:** Jonny Coppel, Vassiliki Bountziouka, Daniel Martin, Edward Gilbert-Kawai

**Affiliations:** 1 University College London Centre for Altitude Space and Extreme Environment Medicine, UCLH NIHR Biomedical Research Centre, Institute of Sport Exercise and Health, 170 Tottenham Court Road, London, W1T 7HA UK; 2 Statistical Support Service, Population, Policy and Practice Programme, Institute of Child Health, University College London, London, England; 3 University College London Division of Surgery and Interventional Science, Royal Free Hospital, London, NW3 2QG UK

**Keywords:** Microcirculation, Microscopy, Validation, Capillary

## Abstract

Sidestream dark field (SDF) imaging enables direct visualisation of the microvasculature from which quantification of key variables is possible. The new MicroScan USB3 (MS-U) video-microscope is a hand-held SDF device that has undergone significant technical upgrades from its predecessor, the MicroScan Analogue (MS-A). The MS-U claims superior quality of sublingual microcirculatory image acquisition over the MS-A, however, this has yet to be robustly confirmed. In this manuscript, we therefore compare the quality of image acquisition between these two devices. The microcirculation of healthy volunteers was visualised to generate thirty video images for each device. Two independent raters, blinded to the device type, graded the quality of the images according to the six different traits in the Microcirculation Image Quality Score (MIQS) system. Chi-squared tests and Kappa statistics were used to compare not only the distribution of scores between the devices, but also agreement between raters. MS-U showed superior image quality over MS-A in three of out six MIQS traits; MS-U had significantly more optimal images by illumination (MS-U 95% optimal images, MS-A 70% optimal images (p-value 0.003)), by focus (MS-U 70% optimal images, MS-A 35% optimal images (p-value 0.002)) and by pressure (MS-U 72.5% optimal images, MS-A 47.5% optimal images (p-value 0.02)). For each trait, there was at least 85% agreement between the raters, and all the scores for each trait were independent of the rater (all p-values > 0.05). These results show that the new MS-U provides a superior quality of sublingual microcirculatory image acquisition when compared to old MS-A

## Background

Sublingual video-microscopy is becoming an increasingly important clinical technique used for real-time assessment of the in-vivo microcirculation [1]. The technology permits evaluation of several variables including vessel density, perfusion indices (such as the proportion of perfused vessels and microvascular flow index), and the heterogeneity of the blood flow throughout the capillary bed. Through measuring these variables, sublingual video-microscopy directly quantifies the microcirculation, and this is essential given that it can bear no resemblance to common ‘macro-circulation’—variables such as blood pressure which we usually quantify and then make microcirculatory inferences from [2]. Additionally, studies have shown it is possible to measure variables related to leucocytes, including quantity and kinetics [3–5]. In light of this, video-microscopy therefore offers the potential to optimize treatment of the microvasculature, particularly fluid management and inotropic support in critically ill patients [6].

Since the advent of orthogonal polarisation spectroscopy in 1971 [7] numerous methods have been developed to illuminate the microcirculation including sidestream- (SDF) [8] and incident- dark field imaging (IDF) [9]. The technique exploits the process of incident dark field illumination, whereby blood vessels < 100 µm in diameter, and < 1000 µm below the surface of the organ, are illuminated and visualised in a two-dimensional plane. Both SDF and IDF illuminate the microcirculation using a series of concentrically placed light emitting diodes (LEDs) surrounding a central light guide that contains the lens system. This structure optically isolates the lens from the illuminating outer ring of LEDs, thus preventing contamination of the image with tissue surface reflections [7]. Pulsed green light (wavelength 540 ± 10 nm) that is in synchrony with the video camera frame rate, performs intra-vital stroboscopy, with short illumination times used to help to prevent the smearing of moving objects such as flowing red cells, and the motion-induced blurring of capillaries [10].

**Fig. 1:**
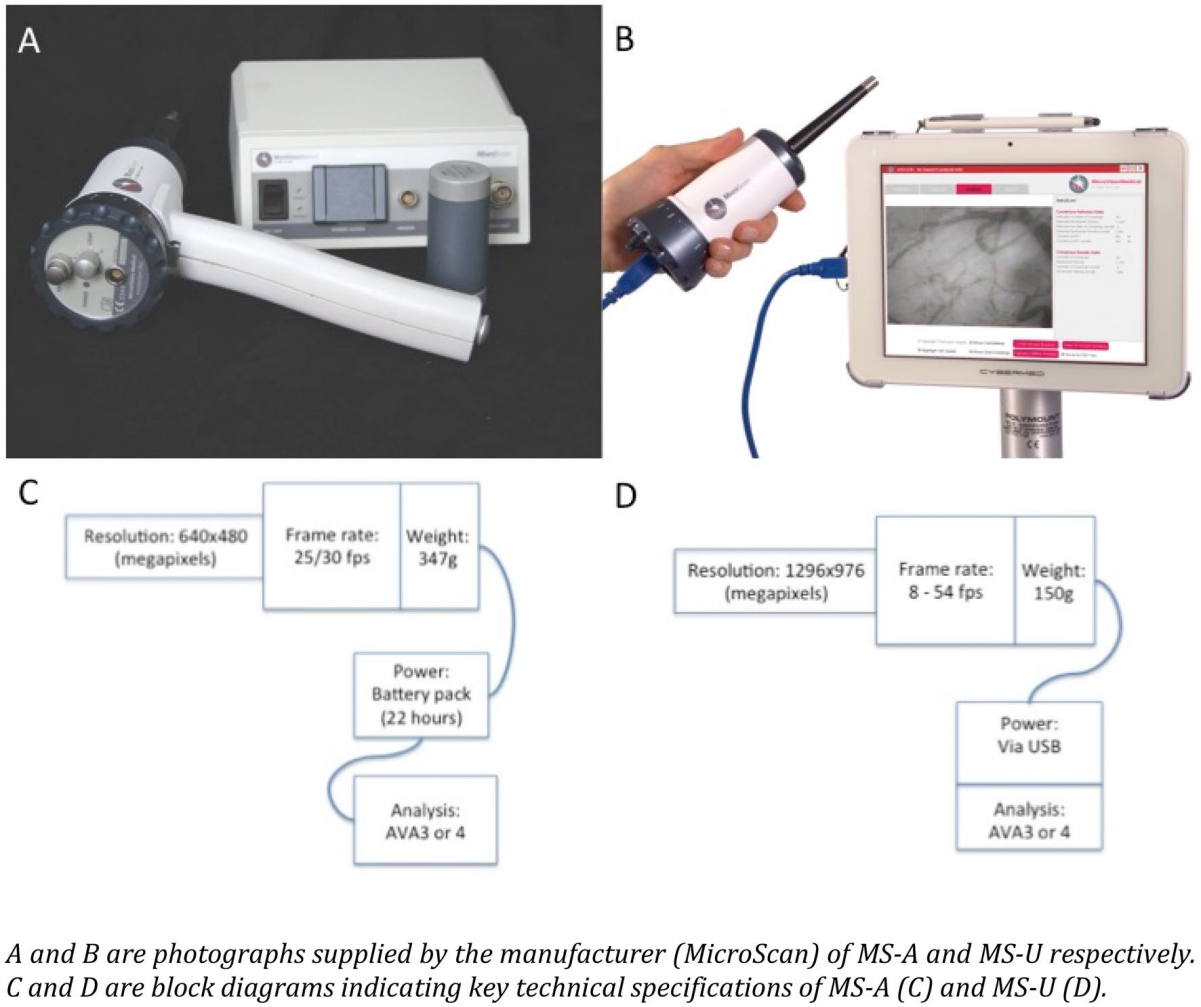
Photographs of the cameras and block diagrams outlining the key differences

The first SDF camera, the MicroScan Analogue (MS-A), was released by Microvision Medical, (Amsterdam, The Netherlands) in 2007. In 2012, Braedius Medical (Huizen, The Netherlands) introduced a new sublingual video-microscope—the Cytocam IDF, and this demonstrated significantly superior image acquisition when compared to the MS-A [11]. In 2018, Microvision Medical revealed their new and updated version, the MicroScan USB3 (MS-U), claiming an improved quality of the data acquisition compared to their earlier model—the MS-A. The updated camera has a number of objective improvements compared to its predecessor (see Table 1; Fig. 1), including a higher camera resolution, an increased frame rate, a much lower weight (predominantly due to its custom built camera as opposed to its predecessors use of a bulkier third-party camera), and a conversion from analogue to digital image capture. Although these improvements would imply that the MS-U should demonstrate significant superiority in terms of the quality of image acquisition over its predecessor, this has not been validated and requires confirmation. This study therefore directly compares the upgraded 2018 Microvision MS-U camera with the previous 2007 analogue MS-A model.

**Table 1:** A comparison of properties between MicroScan analog and MicroScan USB3

Property	MicroScan analog (MS-A)	MicroScan USB3 (MS-U)
Magnification	5 ×	5 ×
Optical resolution	2.1 µm	2.1 µm
Field of view (mm)	0.94 × 0.75	0.94 × 0.75
Camera resolution (megapixels)	0.3 (640 × 480)	1.3 (1296 × 976)
µm / pixel	1.5 × 1.6	0.7 × 0 .8
Frame rate (fps)	25/30	8–54 (adjustable in 1 frame/s steps in AVA 4.x)
Illumination	6xLED (540 nm)	6 x LED (540 nm)
Pulse duration (ms)	16	0–16 (adjustable in 62.5µs steps in AVA 4.x)
Weight (g)	347	150
Power supply	Battery pack (22 h)	USB powered
Analysis	AVA3 or AVA4	AVA 4. ×
Interface	BNC	USB3

## Methods

Ethical approval for the study was obtained from University College London Research and Ethics Committee. A total of sixty videos (30 for each device), were obtained from healthy volunteers who had given informed consent. The data capture was carried out in a single laboratory (London, UK). Volunteers rested for ten minutes in the supine position before images were obtained whereby the investigator positioned and focused the cameras under the participants’ tongue. Ten seconds of video footage were digitally recorded onto the computer, where images were stored for later analysis. This process was repeated on each participant until six good quality recordings, three from each device, had been acquired from separate areas of the sublingual region. The order of use of the device was randomly generated. All images were obtained by one of two researchers, both of whom were experienced in using the video microscopes. The videos were taken according to the new video-microscopy consensus guidelines [12].

After video acquisition, the videos were saved onto a hard drive and were then reviewed on the same computer using Windows Media Player (Microsoft Corporation, Washington, US) without any pre-processing. Two raters (JC, EGK) blinded to the device on which the video file was recorded, independently graded the films according to the Microcirculation Image Quality Score (MIQS) system [13] (Table 2). With this semi-objective approach to grading the quality of image acquisition prior to analysis, each of the six categories is graded as 0 (optimal), 1 (acceptable) or 10 (unacceptable). If the total of the six categories is > 10, then the video is unsuitable for analysis.

**Table 2:** The Microcirculation Image Quality Score

Category	Brief description	Optimal (0)	Acceptable (1)	Unacceptable (10)
Illumination	Brightness and contrast of video	Even illumination across the entire field of view. Contrast sufficient to see small vessels against a background of tissue	The video borders on being too dark or bright to distinguish vessels from tissue but the vessels are still identifiable.	The video is oversaturated/too bright or too dark to make out analysable features. Insufficient contrast to resolve flow rate
Duration	Number of frames in the video clip and how it represents the actual pathology	Analysable video segment is ≥ 5 s long (> 150 frames)	Analysable video segment is 3–5 s (between 90 and 150 frames)	Analysable video segment < 3 s (90 frames)
Focus	Image sharpness in region of interest	Good focus for all vessels (small and large) in the entire field of view. Plasma gaps and red blood cells are visible	< 1/2 field of view is out of focus or edges of the vessels are slightly out of focus	Video is completely out of focus such that no small vessel can be seen
Content	Determination of the types of vessels and/or presence of occluding artefacts in the image	Video is free of occlusions. Good distribution of large and small vessels. Less than 30% of the vessels are looped upon themselves	Video may have a few artefacts. Acceptable distribution of large and small vessels. About 30–50% of the vessels are looped	Most of the field of view has occluding artefacts such as saliva or bubbles. More that 50% vessels are looped upon themselves
Stability	Frame motion that can be adequately stabilised without motion blur	Movement is within 1⁄4 of the field of view. No motion blur	Movement is within 1⁄2 field of view. No motion blur	Movement is greater than 1⁄2 of the field of view and/or motion blur in frame
Pressure	Iatrogenic mechanical pressure causing misrepresentation of flow	Flow is constant throughout the entire movie. No obvious signs of artificially sluggish or stopped flow. Good flow in the largest vessels	Signs of pressure (localised sluggish flow in a specific large vessel), but flow appears to be unimpeded based on good flow in most large vessels	Obvious pressure artefacts associated with probe movement, and/or flow that starts and stops, reversal of flow. Poor or changing flow in larger venules

Adapted from ‘Quality Scoring Metrics: The microcirculation image quality score: development and preliminary evaluation of a proposed approach to grading quality of image acquisition for bedside videomicroscopy [10]

Chi-squared tests were used to determine whether scores (optimal and acceptable) for each trait were independent of the Rater and of the Video-microscope. Agreement between Raters and agreement between Video-microscopes were assessed using Kappa statistic. Agreement was not due to chance for values of Kappa statistic > 0.60. The two-tailed significance level was set at 0.05, and R(version 3.4.3) was used for the analyses.

## Results

**Fig. 2:**
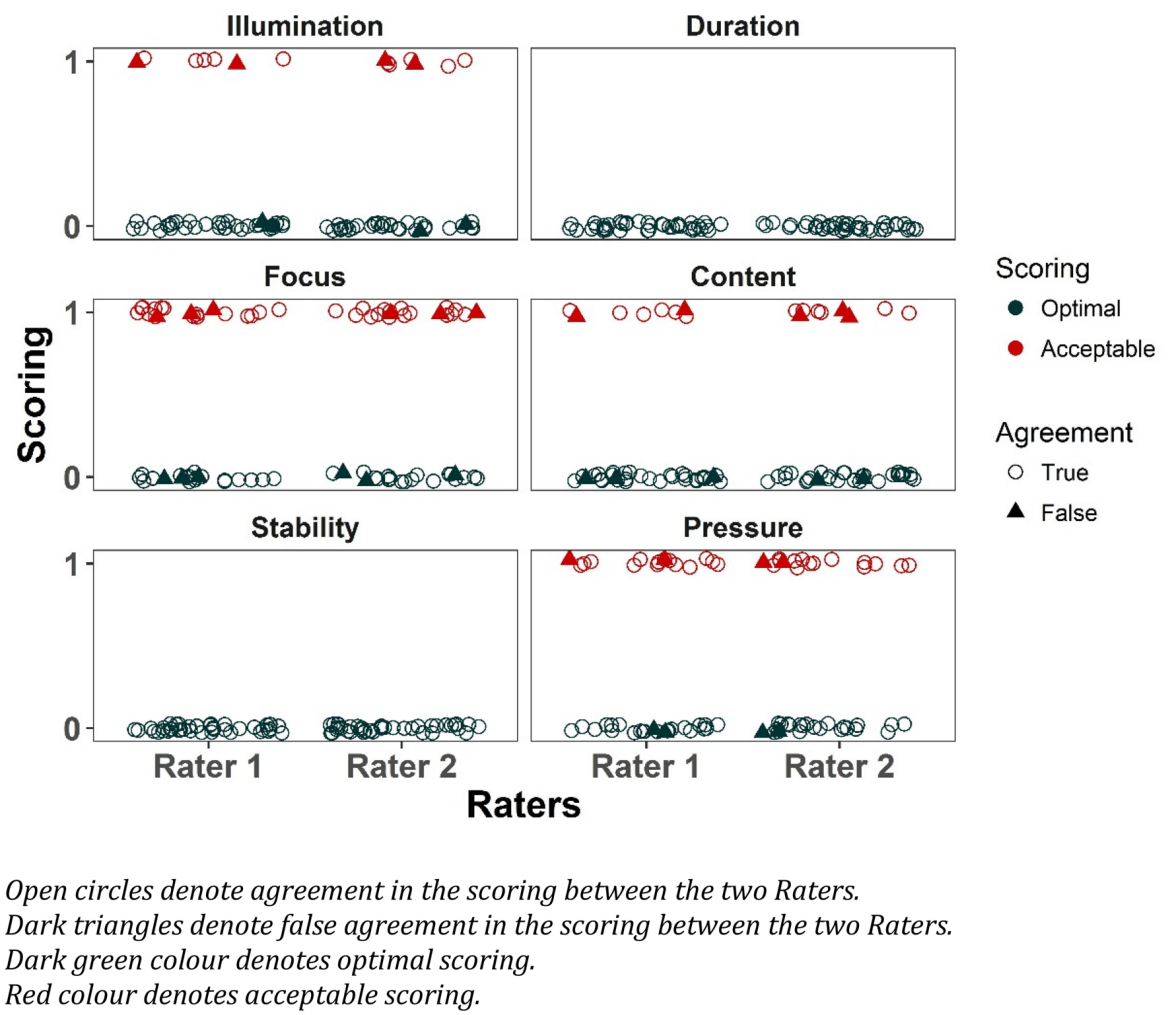
Film scores distributed by rater for each category

All 60 videos were analysed by both raters, and no problems were encountered. The distribution of scores by rater is shown in Fig. 2.

**Fig. 3:**
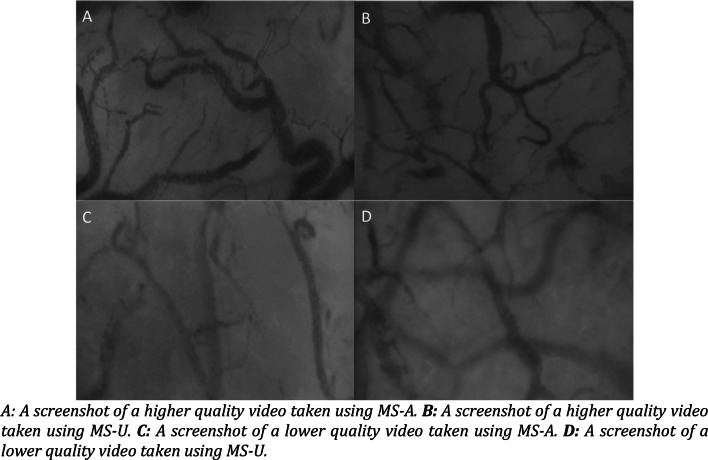
Example screenshots of higher and lower quality images taken from both devices

MS-U was rated as having superior image quality over MS-A in three of out six MIQS traits (Table 2). MS-U captured significantly more optimal images in terms of; (i) illumination (MS-U 95% optimal images, MS-A 70% optimal images (p-value 0.003)); (ii) focus (MS-U 70% optimal images, MS-A 35% optimal images (p-value 0.002)); and (iii) pressure (MS-U 72.5% optimal images, MS-A 47.5% optimal images (p-value 0.02)). There was no significant difference between the content capture of the two video-microscopes (MS-U 77.5% optimal images, MS-A 80% optimal images (p-value 0.79)), and both techniques demonstrated 100% optimal images acquisition in terms of duration and stability (Table 3). Please see Fig. 3 for example screenshots of higher and lower quality videos taken using MS-U and MS-A.

**Table 3:** Distribution of scores by device for each category

	MS-U	MS-A	p
Illumination, n			0.003
Optimal (%)	38 (95)	28 (70)	
Acceptable (%)	2 (5)	12 (30)	
Duration, n (%)*			–
Optimal (%)	40 (100)	40 (100)	
Acceptable (%)	0 (0)	0 (0)	
Focus, n (%)			0.002
Optimal (%)	28 (70)	14 (35)	
Acceptable (%)	12 (30)	26 (65)	
Content, n (%)			0.79
Optimal (%)	31 (77.5)	32 (80)	
Acceptable (%)	9 (22.5)	8 (20)	
Stability, n (%)*			–
Optimal (%)	40 (100)	40 (100)	
Acceptable (%)	0 (0)	0 (0)	
Pressure, n (%)			0.02
Optimal (%)	29 (72.5)	19 (47.5)	
Acceptable (%)	11 (27.5)	21 (52.5)	

*Both raters gave the same scores for duration and stability therefore there was no variability for a Kappa statistic to be calculated

Agreement between the two raters was good, as evidenced by being 85% or over for each trait tested, and all kappa values were over 0.60 demonstrating these results were not due to chance (Table 4). Additionally the scores for each trait were independent of the rater (all p-values > 0.05) (Table 4).

**Table 4:** Agreement between raters for each of the categories

	Agreement (%)	Kappa	Std. Err.	p
Illumination	90	0.65	0.16	< 0.001
Duration*	100	–		
Focus	85	0.70	0.16	< 0.001
Content	88	0.63	0.16	< 0.001
Stability*	100			
Pressure	90	0.79	0.16	< 0.001

*Both raters gave the same score for the duration and stability, so given the lack of variability, the kappa statistic cannot be calculated

## Discussion

These results demonstrate for the first time, that the MS-U video microscope is superior to MS-A video microscope in terms of the quality of image acquisition. The agreement between the raters on each MIQS trait was at least 85% with Kappa statistics of over 0.63, a positive indicator of the reliability of the study. Using the total score value to determine if an image was deemed suitable or unsuitable for analysis, there was 100% agreement between the two raters. The categories of the MIQS that showed the greatest difference between the two cameras were illumination, focus and pressure. The former two may be as a result of the new illumination management system, and also the improved optical resolution of the MS-U. The improvement in pressure scoring may be because the MS-U device is lighter and therefore less prone to a pressure artifact. No difference was seen in the duration, stability and content of image capture, however, this is unsurprising given that these traits are generally independent of the device used. Duration and stability, were 100% optimal across both devices. This is likely to have been because these two traits in particular are less dependent on the device being used, but more dependent on the person capturing the images, and the subject’s anatomy and degree tongue movement. Additionally, in the updated MS-U, the software stops filming after a specific time frame, and this can be preset prior to image capture.

Whilst this study found significant differences between MS-U and MS-A, the Cytocam IDF video-microscope has also been shown to be superior to the MS-A [11]. Unfortunately it is not possible to make comparisons between the Cytocam IDF and MS-U using these two independent studies, however, one contrasting feature of this study compared to the Cytocam IDF vs. MS-A study, is that no videos in this study were scored as unacceptable [11]. A future study directly comparing the Cytocam IDF and MS-U is therefore warranted, as results obtained from video-microscopy assessment of the microcirculation fundamentally rely on optimal image capture [13].

Strengths of this paper include the agreement witnessed between the two raters and the size of the p-values demonstrated in the results, with all significant p-values being at least 0.02 or below. Limitations are also evident, perhaps the foremost being that the MIQS still relies on subjective rater assessment of the videos. This is however, still the gold-standard approach for grading images of the microcirculation prior to variable analysis. Another limitation is that we have only compared these video-microscopes, on one capillary bed location in the body. Although the sublingual microvasculature is currently the most widely investigated, further work involving other capillary beds should use the results of this study with caution.

Notably further studies should be considered regarding video-microscopy image acquisition and analysis. Whilst this study has solely measured and compared the quality of image acquisition between two devices, it has not considered the recently developed automated analysis software that has been validated using IDF [14], enabling automated processing of the images, thus providing objective figures such as microcirculatory flow index. Of note, however, this software is reliant on high image quality to work [14]. As manual image analysis is both subjective in nature, and a very time consuming process, automated analysis is the key to enabling sublingual video microscopy to be used at the bedside in a clinical setting. The software has however yet to be validated for this new SDF device, and future studies should seek to do this.

## Conclusions

In this study we have established that the latest MicroVision SDF video-microscope demonstrates superior image acquisition when compared to its predecessor. In three out of six MIQS categories -illumination, image focus and avoidance of pressure artifacts, the MS-U out-performed the MS-A. The findings therefore support the claims made by the manufacturers claiming superior image acquisition over the MS-A. With its optimal degree of image capture, the MS-U better portrays the underlying sublingual microcirculation, and should therefore be used for its real-time assessment.

## List of abbreviations

µm: Micrometer
IDF: Incident dark field
LED: Light emitting diode
MIQS: Microcirculation Image Quality Score
MS-A: Microscan Analogue
MS-U: Microscan USB3
SDF: Sidestream dark field
